# Use of an electronic early warning score and mortality for patients admitted out of hours to a large teaching hospital

**DOI:** 10.1186/cc14488

**Published:** 2015-03-16

**Authors:** J Bannard-Smith, S Abbas, S Ingleby, C Fullwood, S Jones, J Eddleston

**Affiliations:** 1 Manchester Royal Infirmary, Manchester, UK

## Introduction

There is widespread concern regarding excess mortality for patients admitted to hospital out of hours. We introduced an electronic track and trigger system (Patientrack) with automated alerts in a large university teaching hospital between 2010 and 2012. The system computes the patient's early warning score and alerts medical staff via a pager. It is operational 24 hours a day, 7 days a week and could be an effective tool to reduce variations in mortality throughout the working week.

## Methods

We extracted hospital outcome data for all admissions during the financial years between 2007 and 2014. We identified variables that predicted mortality and incorporated them into a multivariate logistic regression model to assess risk of death for admissions in hours (9:00 am to 5:00 pm, Monday to Friday) versus out of hours (all other times).

## Results

Data were available for 1,180,268 hospital admissions, of which 7,264 (0.6%) died. Predictors for hospital mortality included: age, male sex, unplanned admission and admission from supportive care. Risk of death for out-of-hours admissions was not significantly different to in-hours for any year (1.01 (0.92 to 1.11), *P *= 0.784). There was a significant fall in risk of death over the 7-year period compared with baseline values in 2007/08 (Table 1).

**Table 1:** Overall risk of death ratios compared to 2007/08.

Year	ROD	95% CI
2008/09	1.01	0.73 to 1.5
2009/10	1.03	0.74 to 1.5
2010/11	0.46*	0.33 to 0.7
2011/12	0.31*	0.22 to 0.4
2012/13	0.27*	0.2 to 0.39
2013/14	0.26*	0.2 to 0.37

^*^P < 0.001.

## Conclusion

In our cohort there was no evidence of increased mortality for patients admitted out of hours compared with in hours. This remained true after adjustment for age, sex, emergency admissions and admission source. Our data demonstrated an overall fall in risk of death over the study period. The Introduction of Patientrack could have contributed to this reduction in mortality.

